# Hepatic–vascular crosstalk via GRK2: fenofibrate improves endothelial function by restoring lipid metabolism and NO signaling in obese mice

**DOI:** 10.3389/fphys.2025.1734746

**Published:** 2026-01-12

**Authors:** Kumiko Taguchi, Haruka Yonaiyama, Tomoya Furukawa, Takayuki Matsumoto, Tsuneo Kobayashi

**Affiliations:** 1 Department of Physiology and Morphology, Institute of Medicinal Chemistry, Hoshi University, Shinagawa-ku, Tokyo, Japan; 2 Second Department of Pharmacology, School of Pharmaceutical Sciences, Kyushu University of Medical Science, Nobeoka, Japan

**Keywords:** endothelial dysfunction, fenofibrate, hepatic GRK2, NO production, obesity

## Abstract

**Background:**

Obesity is often linked to endothelial dysfunction, a key factor in the development of cardiovascular and metabolic diseases. Reduced nitric oxide (NO) bioavailability is a defining feature of this condition, yet its underlying mechanisms and possible therapeutic targets remain unclear. Fenofibrate, a peroxisome proliferator-activated receptor-α (PPARα) agonist, is widely used to regulate lipid metabolism; however, its influence on vascular function and associated molecular pathways is not fully established. This study examined the effects of fenofibrate on vascular reactivity in high-fat diet (HFD)-induced obese mice, focusing on endothelial NO production and its upstream regulators.

**Methods:**

Male Institute of Cancer Research mice were fed either a standard diet (SD) or an HFD for 12 weeks. Two weeks before the end of the feeding period, mice were treated with fenofibrate (25 mg/kg/day) or vehicle, forming four groups: SD, SD with fenofibrate (SD-FF), HFD, and HFD with fenofibrate (HFD-FF). Lipid profiles, aortic vascular function, and NO production were evaluated. Phosphorylation levels of liver kinase B1 (LKB1), AMP-activated protein kinase (AMPK), and Akt were analyzed, along with G protein-coupled receptor kinase 2 (GRK2) expression and activity in the aorta and liver.

**Results:**

HFD-FF mice showed markedly lower hepatic and plasma triglyceride levels than HFD mice, indicating improved lipid metabolism. Endothelial-dependent relaxation, which was impaired in HFD mice, was markedly restored in HFD-FF mice, accompanied by increased basal NO production. Aortic phosphorylation of LKB1, AMPK, and Akt was enhanced in HFD-FF mice relative to HFD mice, whereas aortic GRK2 activity remained unchanged. In the liver, GRK2 expression was elevated in HFD and HFD-FF groups compared with SD mice, but GRK2 activity was markedly increased in HFD mice and notably reduced in HFD-FF mice.

**Conclusion:**

Fenofibrate improves endothelial-dependent relaxation and NO production in HFD-induced obese mice, likely through activation of the LKB1/AMPK/Akt pathway. The suppression of hepatic GRK2 activity by fenofibrate may contribute to better lipid metabolism, thereby promoting the recovery of vascular function.

## 1 Introduction

Obesity is a major risk factor for dyslipidemia, type 2 diabetes mellitus (T2DM), atherosclerosis, hypertension, cardiovascular disease (CVD), and several types of cancer in high-income countries (Evans et al., 2004; Watanabe et al., 2010). A high-fat diet (HFD) is widely recognized as a key contributor to obesity, which is now understood as a chronic, low-grade inflammatory condition driven by proinflammatory mediators secreted from adipose tissue (Khanna et al., 2022). Beyond its link to traditional cardiovascular risk factors, obesity contributes to the development of endothelial dysfunction (Pontiroli et al., 2006). The vascular endothelium and its secreted factors play a crucial role in regulating vascular tone and overall vascular homeostasis (Flammer et al., 2012; Favero et al., 2014). Endothelial dysfunction is primarily characterized by reduced nitric oxide (NO) bioavailability (De Vriese et al., 2000). HFD-induced obesity serves as a well-established experimental model for studying metabolic disturbances and vascular endothelial impairment (Buettner et al., 2007). Chronic HFD feeding results in endothelial dysfunction—a hallmark of early-stage CVD—mainly due to oxidative stress and inflammation that impair NO bioavailability (Kim et al., 2008; Engin, 2017).

Within the vascular endothelium, the Akt signaling pathway plays a central role in NO production (Fleming and Busse, 2003) and is regulated by upstream modulators such as AMP-activated protein kinase (AMPK) and G protein-coupled receptor kinase 2 (GRK2).

AMPK performs multiple biological functions, including the regulation of glucose and lipid metabolism as well as endothelial NO synthase (eNOS) expression (Ceolotto et al., 2007; Lee and Kim, 2010). Modulation of AMPK is a promising therapeutic target for metabolic disorders such as obesity (Hardie, 2018). AMPK activation is primarily mediated by the upstream kinase liver kinase B1 (LKB1) (Woods et al., 2003), which forms a heterotrimeric complex with regulatory proteins essential for its activation and cytosolic localization (Boudeau et al., 2003). Additionally, García-Prieto et al. (2015) demonstrated that HFD-induced endothelial dysfunction in mice is associated with reduced AMPK activity, the activation of which enhances endothelial NO bioavailability (García-Prieto et al., 2015).

GRK2 has emerged as a critical regulator of cardiovascular function (Penela et al., 2003; Ciccarelli et al., 2011; Santulli et al., 2013). Elevated GRK2 expression and activity have been observed in metabolic disorders, including obesity and T2DM (Taguchi et al., 2011; 2012a; 2012b; 2017), suggesting that GRK2 inhibition may help restore endothelial function. GRK2 suppresses Akt/eNOS-mediated NO production in diabetic vascular models (Taguchi et al., 2011; 2012a; 2012b), whereas hepatic GRK2 downregulation or inhibition improves endothelial-dependent relaxation by rescuing Akt/eNOS signaling (Taguchi et al., 2017). However, evidence regarding how fenofibrate affects LKB1/AMPK/Akt and GRK2/Akt signaling in vascular endothelial function under obese conditions remain limited.

Fenofibrate, a fibric acid derivative and peroxisome proliferator-activated receptor-α (PPARα) agonist, is primarily known for increasing high-density lipoprotein cholesterol (HDL-C) and reducing triglyceride (TG) levels (Kon et al., 2004; Khera and Plutzky, 2013). Beyond its lipid-modulating effects, fenofibrate exhibits pleiotropic vasculoprotective properties, including antioxidant (Wang et al., 2010; Walker et al., 2012; Price et al., 2012; Krysiak et al., 2013), antithrombotic (Undas et al., 2006; Lee et al., 2009), and antiapoptotic actions (Zanetti et al., 2008; Tomizawa et al., 2011). However, the mechanisms underlying its vascular benefits, particularly in diabetes, are not fully understood.

In this study, we investigated the effects of fenofibrate on vascular endothelial function in HFD mice, particularly focusing on the potential involvement of GRK2. Considering the critical role of the LKB1/AMPK/Akt signaling pathway in regulating endothelial NO production and its modulation by GRK2 activity, we aimed to determine whether fenofibrate-mediated improvement in endothelial function is associated with suppression of hepatic GRK2 overactivation and restoration of LKB1/AMPK/Akt pathway activity in this model.

## 2 Materials and methods

### 2.1 Animal experimentation

Male Institute of Cancer Research (ICR) mice (4 weeks old) were obtained from the Tokyo Animal Laboratories (Tokyo, Japan). All mice were randomly divided into cages (n = 5 per cage) and received a sterile rodent chow diet (MF; Oriental Yeast Co., Ltd, Tokyo, Japan) and water *ad libitum*. They were designated the SD group. At 4 weeks of age, some mice were given a sterilized HFD (D12492 with 60% energy from fat and 0.03% w/w cholesterol; Research Diets, New Brunswick, NJ, United States) for an additional 12 weeks, which were designated the HFD group. Some mice in the SD or HFD group were injected with carrier solution (dimethyl sulfoxide), and the other mice in the treated groups were injected with fenofibrate (FF; 25 mg/kg/day, i.p.; Cayman Chemical, Ann Arbor, MI, United States). They were designated the SD-FF group or the HFD-group. The injection was performed daily for 14 days while keeping animals on MF or HFD. The mice were anesthetized, and the samples, including the plasma, thoracic aortas, and livers, were collected and used for further analyses. Procedures were performed in accordance with the principles and guidelines on animal care of Hoshi university Animal Care and Use Committee as reviewed by the Ethics Committee (P23-043) (accredited by the Ministry of Education, Culture, Sports, and Science and Technology of Japan).

### 2.2 Measurement of plasma and liver parameters

Blood taken from the abdominal aorta at death was collected and subjected to centrifugation at 4 °C at 3,500 rpm for 10 min, followed by plasma collection. Plasma samples were analyzed for glucose, total cholesterol, TG, and non-esterified fatty acid (NEFA) concentrations using commercial kits (FUJIFILM Wako Pure Chemical Corporation, Osaka, Japan). For the determination of liver cholesterol and TG levels, 200-mg frozen liver was used to extract lipids according to the methods proposed by Jayaprakasam et al. (2006). Total cholesterol and TG levels were quantified as above. Results were then normalized to the weight of the sample. At that time, the mice were not starved, and samples were taken after the mice had eaten and drunk *ad libitum*.

### 2.3 Measurement of aortic vasoactive responses

The thoracic cavity was opened, and the thoracic aorta was excised and placed in ice-cold modified Krebs–Henseleit solution (KHS; 118 mM NaCl, 4.7 mM KCl, 25 mM NaHCO_3_, 1.8 mM CaCl_2_, 1.2 mM NaH_2_PO_4_, 1.2 mM MgSO_4_, and 11 mM glucose). Excess connective and adipose tissue were removed, and the aorta was cut into 2-mm ring segments. Each segment was mounted under a resting tension of 1.5 g in a 10-mL organ bath containing oxygenated (95% O_2_ and 5% CO_2_) KHS at 37 °C. Aortic tension was recorded using an isometric force transducer, and data were analyzed with LabChart 8 sofware (Dunedin, New Zealand). The measurement protocol was adapted from Taguchi et al. (2011), Taguchi et al. (2012a), Taguchi et al. (2012b), Taguchi et al. (2017). Briefly, mounted aortic rings were stabilized under a resting tension of 1.5 g during which time the KHS in the organ bath was changed every 15 min for 45 min with pre-warmed KHS. Following this, all aortic rings were pre-contracted with prostaglandin F_2α_ (PGF_2α_; 10^–6^ – 3 × 10^−6^ M) (Fuji pharma, Tokyo, Japan) administration to the organ bath until the 1-g contraction tension was reached. Subsequently, aortic rings were randomly divided into groups that either received cumulative concentrations of the known pro-vasodilatory agent, acetylcholine (ACh, 10^–9^ – 10^–5^ M, Daiichi Sankyo Co., Ltd.), sodium nitroprusside (SNP, 10^–10^ – 10^–5^ M, Wako) or clonidine (Sigma-Aldrich, St. Louis, MO, United States). The concentration of PGF_2α_ was adjusted for each preparation to produce a uniform precontraction level (∼1 g) before initiating relaxation assays. This normalization enabled assessment of vasorelaxation independently of intrinsic differences in contractile responsiveness. To examine the involvement of Akt- and AMPK-dependent pathways, pharmacological inhibitors were administered prior to the vasorelaxation experiments. A selective Akt inhibitor (Akt inh; 10^–6^ M; CALBIOCHEM, San Diego, CA, United States, #124005) or the AMPK inhibitor Compound C (CC; 5 × 10^−6^ M; CALBIOCHEM, #171260) was added directly to the organ bath 30 min before incubation of the PGF_2α_-mediated precontraction, followed by ACh- or clonidine-mediated relaxation. Both inhibitors remained in the bath for the duration of the experimental protocol.

### 2.4 Measurement of NO levels

**FIGURE 1:**
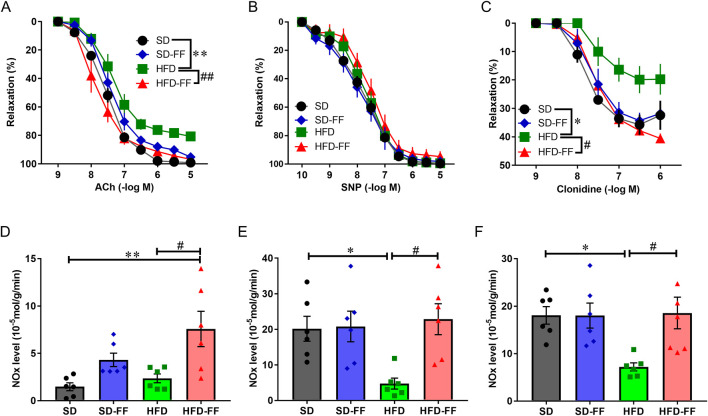
Concentration response curves and NO levels to ACh and SNP and clonidine in aortic rings. **(A)** Acetylcholine (ACh)-induced endothelial relaxation after PGF_2α_ precontraction in aorta of SD (control; standard diet), SD-FF (fenofibrate-treated control), HFD (high-fat diet), and HFD-FF (fenofibrate-treated HFD) mice (n = 5, mean ± SE). **(B)** sodium nitroprusside (SNP)-induced relaxation after PGF_2α_ precontraction in the aorta of SD, SD-FF, and HFD mice (n = 5, mean ± SE). **(C)** Clonidine-induced relaxation after PGF_2α_ precontraction in the aorta of SD, SD-FF, HFD, and HFD-FF mice (n = 5, mean ± SE). **(D)** Levels of NO under nonstimulation (for 20 min) in aortic tissues from SD, SD-FF, HFD, and HFD-FF (n = 6, mean ± SE). **(E)** Levels of NO under ACh-stimulation (10^–6^ M, for 20 min) in aortic tissues from SD, SD-FF, HFD, and HFD-FF (n = 6, mean ± SE). **(F)** Levels of NO under clonidine-stimulation (10^–6^ M, for 20 min) in aortic tissues from SD, SD-FF, HFD, and HFD-FF (n = 6, mean ± SE). *p < 0.05; **p < 0.01 vs. SD. ^#^p < 0.05; ^##^p < 0.01 vs. HFD.

Experiments were conducted as described previously (Taguchi et al., 2011; Taguchi et al., 2012a; Taguchi et al., 2012b; Taguchi et al., 2017). Aortic rings were incubated with basal (nonstimulated), ACh (10^–6^ M), or clonidine (10^–6^ M) for 20 min and then rapidly dried and weighed. The incubation solution (KHS) was analyzed for NO using an ENO-20 NOx Analyzer (Eicom, Kyoto, Japan) according to the manufacturer’s instructions. Basal NO production was measured first, and ligand-induced NO production was calculated by subtracting basal values from the NO concentration obtained during ACh or clonidine stimulation. Thus, the NO values shown in Figures 1E,F represent ligand-dependent NO generation. Because the objective was to assess agonist-driven endothelial NO synthesis, PGF_2α_ was omitted during NO measurements, consistent with established protocols for agonist-specific NO assays.

### 2.5 Western blotting

Total proteins were extracted from mouse aortic tissues. Because FF notably increased basal NO production in HFD mice, protein analyses were conducted under unstimulated conditions to investigate the chronic mechanisms underlying this enhancement. Phosphorylation of LKB1, AMPK, and Akt was assessed in whole aortic lysates. Because the tissue contains endothelial and smooth muscle cells, these results reflect signaling activity across mixed vascular cell populations. The proteins were denatured and then electrophoresed on polyacrylamide gels with a loading volume of 25-μg protein. The proteins were transferred to polyvinylidene fluoride (PVDF) membranes and then closed in immunoblock for 90 min at room temperature, followed by the addition of primary antibodies. Incubate PVDF membranes at 4 °C overnight. Primary antibodies used were as follows: anti–phospho-LKB1 (#3055, 1:1000; Cell Signaling Technology, Danvers, MA, United States), anti-LKB1 (#GTX130697, 1:1000; GeneTex, Inc., Irvine, CA, United States), anti–phospho-AMPKα (Thr172; CST, #2535, 1:1000), anti-AMPKα (CST, #2532, 1:1000), ant–phospho-Akt (Ser473; CST, #9271, 1:1000), ant-Akt (CST, #9272, 1:1000), anti–phospho-GRK2 (Ser670; GeneTex, Inc., #GTX24473, 1:1000), anti-GRK2 (#sc-13143, 1:1000; Santa Cruz Biotechnology, Dallas, TX, United States), and anti–β-actin (#A5316, 1:10000; Sigma Chemical Co., St. Louis, MO, United States). Secondary antibodies were HRP-conjugated anti-rabbit (#W4011, 1:10000; Promega, Fitchburg, WI, United States) or anti-mouse IgG (#W4021, 1:10000; Promega). Blots were developed with a chemiluminescence detection reagents. Consistent with previous reports, the GRK2 activity was assessed indirectly by quantifying the phosphorylation levels of GRK2 at Ser670, a modification shown to correlate positively with GRK2 functional activation in metabolic and cardiovascular tissues (Taguchi et al., 2011; Taguchi et al., 2012b). Phosphorylation levels were measured by Western blotting and expressed relative to total GRK2 to reflect the activation status.

### 2.6 Analysis and statistics

The percentage of relaxation was calculated by setting the maximum contraction immediately before vasorelaxant addition as 0% and complete recovery to the baseline tension (1.5 g) before PGF_2α_ application as 100%. Curve fitting was performed using GraphPad Prism7 (San Diego, Calif., United States). Data are expressed as the mean ± standard error (SE), with *n* indicating the number of experiments. Datasets involving two independent variables (diet and FF treatment) were analyzed using two-way analysis of variance (ANOVA) followed by Tukey’s *post hoc* test. Concentration–response curves were analyzed using two-way repeated-measures ANOVA followed by Tukey’s test, where appropriate. A *p*-value of <0.05 was considered statistically significant.

## 3 Results

### 3.1 Fenofibrate improved lipid metabolism in HFD-induced obese mice

At 4 weeks of age, ICR mice were fed an HFD *ad libitum* for 12 weeks. Body weight and plasma parameters are shown in Table 1. After 12 weeks on the HFD, mice markedly increased body weight and elevated plasma cholesterol, TG and NEFA levels compared with the SD group. FF administration markedly reduced plasma TG and NEFA levels in HFD mice. However, plasma cholesterol and nonfasting glucose levels did not differ markedly between HFD and HFD-FF mice (Table 1). These findings indicate that FF treatment effectively prevented or mitigated HFD-induced obesity and dyslipidemia.

**TABLE 1:** Body weight and plasma parameters after 2 weeks of fenofibrate treatment in SD and HFD mice.

Items	SD (n = 15)	SD-FF (n = 15)	HFD (n = 15)	HFD-FF (n = 15)
Body weight (g)	44.1 ± 1.3	42.4 ± 1.3	60.1 ± 2.5***	53.5 ± 3.3*
Glucose (mg/dL)	226.7 ± 11.1	218.3 ± 6.6	251.0 ± 14.0	215.2 ± 9.3
Cholesterol (mg/dL)	87.1 ± 5.0	98.0 ± 4.0	131.7 ± 7.2***	122.4 ± 10.3**
Triglyceride (mg/dL)	109.1 ± 11.9	93.5 ± 6.6	162.3 ± 10.8**	119.8 ± 8.2^#^
NEFA (mEq/L)	1.10 ± 0.03	0.81 ± 0.05 ***	1.28 ± 0.04*	0.75 ± 0.06***^###^

Data are expressed as means ± SE.

SD, standard diet; FF, fenofibrate; HFD, high-fat diet; NEFA, non-esterified fatty acid.

*p < 0.05; **p < 0.01; ***p < 0.001 vs. SD, ^###^ *p* < 0.001 vs. HFD.

Compared with SD mice, HFD mice had increased liver weight and hepatic cholesterol and TG contents. FF treatment markedly reduced liver weight and TG levels, but not cholesterol levels in HFD mice (Table 2). These results suggest that FF restored hepatic lipid metabolism primarily by reducing TG synthesis and accumulation rather than by suppressing cholesterol synthesis.

**TABLE 2:** Liver weight and liver cholesterol and triglyceride levels after 2 weeks of fenofibrate treatment in SD and HFD mice.

Items	SD (n = 15)	SD-FF (n = 15)	HFD (n = 15)	HFD-FF (n = 15)
Liver weight (g)	2.3 ± 0.1	2.4 ± 0.1	3.2 ± 0.2**	3.0 ± 0.2*
Cholesterol (mg/g)	2.9 ± 0.1	2.5 ± 0.1	5.4 ± 0.8**	4.9 ± 0.4*
Triglyceride (mg/g)	1.9 ± 0.1	1.7 ± 0.1	3.3 ± 0.2***	2.6 ± 0.2*^#^

Data are expressed as means ± SE.

SD, standard diet; FF, fenofibrate; HFD, high-fat diet.

*p < 0.05; **p < 0.01; ***p < 0.001 vs. SD; ^#^ *p* < 0.05 vs. HFD.

### 3.2 Vascular reactivity and NO production in aortas

We next evaluated the effects of chronic FF administration on vascular reactivity and aortic NO production. Aortas were isolated from SD, SD-FF, HFD, and HFD-FF mice, and relaxation responses to ACh, SNP, and clonidine were examined, along with NO production under ACh or clonidine stimulation. After precontraction with PGF_2α_ reached a plateau, ACh, SNP, or clonidine was cumulatively added. ACh-induced and clonidine-induced relaxations were markedly reduced in aortic rings from HFD mice compared with SD mice (Figures 1A,C). These impaired relaxations were markedly improved by FF administration (HFD-FF mice). In contrast, SNP-induced relaxation did not differ markedly among the four groups (Figure 1B). Although vessels from FF-treated groups exhibited a trend toward reduced contractile responsiveness, the concentration of PGF_2α_ was individually adjusted (1–3 × 10^−6^ M) to ensure that all arterial rings achieved a comparable level of precontraction (∼1 g). This normalization confirms that differences in ACh- and clonidine-induced relaxation among groups were not attributable to variations in the initial contractile tone. Because ACh- and clonidine-induced vasorelaxations are mediated by NO production (Kobayashi et al., 2004; Taguchi et al., 2011; Taguchi et al., 2012b), we measured NO levels (Figures 1D–F). FF administration markedly increased NO production in HFD mice (Figure 1D). Aortic NO production in response to ACh and clonidine was markedly lower in HFD mice than in SD mice (Figures 1E,F), consistent with the attenuated relaxation responses. In contrast, FF treatment markedly enhanced NO production in HFD mice. Ligand-induced NO production was calculated as the increase above basal levels, representing NO generated specifically in response to ACh or clonidine stimulation. As SNP-induced vasorelaxation occurs independently of the endothelium (Kobayashi et al., 2004), these findings suggest that FF enhances endothelial NO-mediated mechanisms, thereby improving HFD-induced endothelial dysfunction.

**FIGURE 2:**
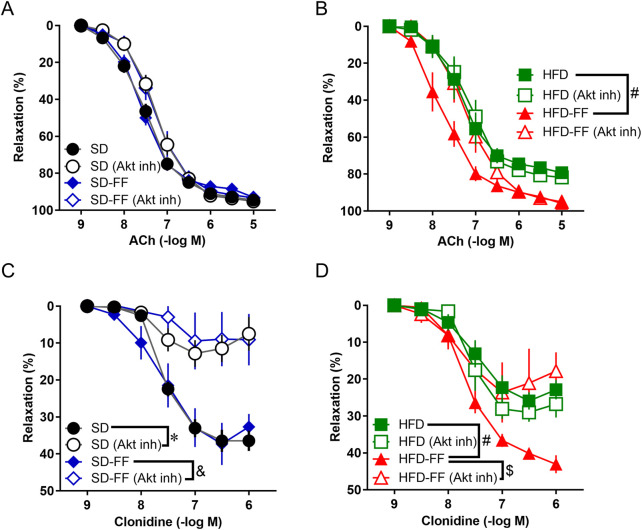
Fenofibrate treatment normalizes vascular relaxation via the Akt pathway in HFD mice. **(A)** Effect of 30-min preincubation with Akt inhibitor (10^–6^ M) on ACh-induced relaxation in the aorta from SD and SD-FF (n = 6, mean ± SE). **(B)** Effect of 30-min preincubation with Akt inhibitor (10^–6^ M) on ACh-induced relaxation in aorta from HFD and HFD-FF (n = 6, mean ± SE). **(C)** Effect of 30-min preincubation with Akt inhibitor (10^–6^ M) on clonidine-induced relaxation in aorta from SD and SD-FF (n = 5, mean ± SE). **(D)** Effect of 30-min preincubation with Akt inhibitor (10^–6^ M) on clonidine-induced relaxation in aorta from HFD and HFD-FF (n = 5, mean ± SE). *p < 0.05 vs. SD. ^&^p < 0.05 vs. SD-FF. ^#^p < 0.05 vs. HFD. ^$^p < 0.05 vs. HFD-FF.

**FIGURE 3:**
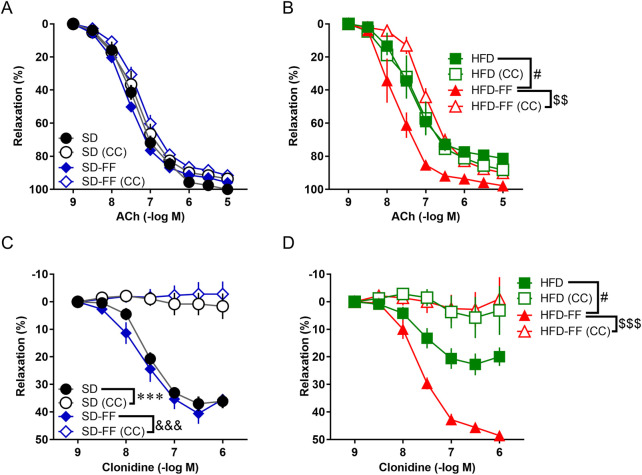
Fenofibrate treatment normalizes vascular relaxation via the AMPK pathway in HFD mice. **(A)** Effect of 30-min preincubation with Compound C (CC; 5 × 10^−6^ M) on ACh-induced relaxation in the aorta from SD and SD-FF (n = 5, mean ± SE). **(B)** Effect of 30-min preincubation with CC (5 × 10^−6^ M) on ACh-induced relaxation in the aorta from HFD and HFD-FF (n = 5, mean ± SE). **(C)** Effect of 30-min preincubation with CC (5 × 10^−6^ M) on clonidine-induced relaxation in the aorta from SD and SD-FF (n = 5, mean ± SE). **(D)** Effect of 30-min preincubation with CC (5 × 10^−6^ M) on clonidine-induced relaxation in the aorta from HFD and HFD-FF (n = 5, mean ± SE). *p < 0.05, ***p < 0.001 vs. SD. ^&&&^p < 0.001 vs. SD-FF. ^#^p < 0.05 vs. HFD. ^$$^p < 0.01, ^$$$^p < 0.001 vs. HFD-FF.

Because only endothelial-dependent relaxation was attenuated by HFD and reversed by FF administration, we next examined the differences in endothelial-dependent signaling pathways triggered by ACh and clonidine. For this purpose, we analyzed the effects of various inhibitors (Figure 2). As shown in Figures 2C,D, the relaxation induced by clonidine was significantly attenuated by an Akt inhibitor. However, the Akt inhibitor did not significantly affect the vascular relaxation induced by ACh (Figures 2A,B). We then investigated the involvement of AMPK. The results showed that the vascular relaxation induced by clonidine almost completely disappeared in the presence of Compound C (an AMPK inhibitor), and Compound C markedly inhibited the ACh-induced relaxation response that was enhanced in the HFD-FF mice (Figure 3). These results suggest that clonidine-induced relaxation response involved NO production via the AMPK/Akt signaling pathway and that FF enhances this pathway. In contrast, ACh-induced relaxation response appeared to promote NO production independently of the AMPK/Akt pathway, whereas FF may enhance this response through an AMPK-dependent mechanism.

### 3.3 Fenofibrate increases NO production via Akt pathway

**FIGURE 4:**
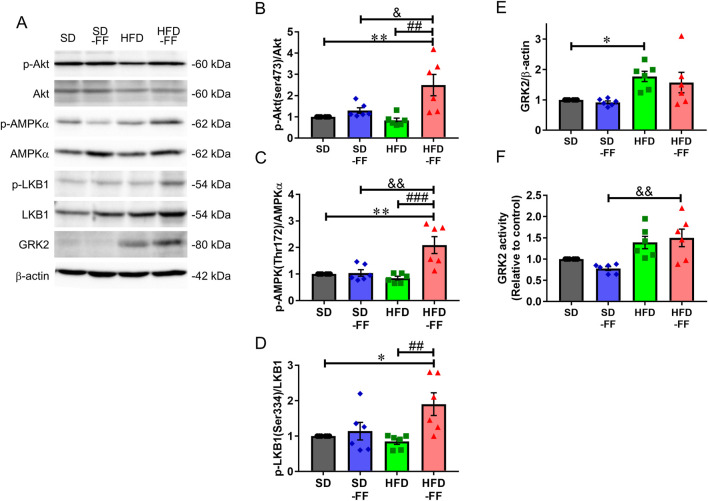
Effect of fenofibrate on regulating the NO production signaling pathway and those related to protein activation in the aorta. The protein levels were measured by Western blot assay in the aortas of SD, SD-FF, HFD, and HFD-FF mice. **(A)** Representative Western blots and quantification of **(B)** p-Akt; **(C)** p-AMPK; **(D)** p-LKB1; **(E)** GRK2 proteins; and **(F)** GRK2 activity (n = 6, mean ± SE). *p < 0.05, **p < 0.01 vs. SD. ^#^p < 0.05, ^##^p < 0.01 vs. HFD. ^##^p < 0.01, ^###^p < 0.001 vs. HFD. ^&^p < 0.05, ^&&^p < 0.01 vs. SD-FF.

Because clonidine-induced relaxation was reduced upon inhibition of the AMPK/Akt/eNOS pathway and this response was impaired in HFD mice but restored by FF, we next analyzed aortic Akt and AMPK phosphorylation across all groups. Total Akt and AMPKα expressions remained unchanged; however, phosphorylated Akt (Ser473) and phosphorylated AMPK (Thr172) levels were markedly increased in HFD-FF mice compared with HFD mice (Figures 4A–C). We also examined the upstream of AMPKα, LKB1. Phosphorylated LKB1 (Ser334) levels were markedly higher in HFD-FF mice compared with SD and HFD mice (Figures 4A,D). We assessed phosphorylation under basal conditions because FF increased NO production even without agonist stimulation (Figure 1D), and defining the mechanism underlying this basal activation was a key aim of the study. FF elevated the basal phosphorylation levels of LKB1, AMPK, and Akt, changes that paralleled the increase in NO production and indicate coordinated activation of these pathways. In SD mice, FF did not markedly alter ACh- or clonidine-induced vasorelaxation, nor did it substantially alter phosphorylation levels of LKB1, AMPK, or Akt. Similarly, the effects of the Akt inhibitor and Compound C were also modest in SD vessels relative to HFD vessels. Collectively, these findings indicate that FF-driven enhancement of Akt-dependent signaling primarily emerges in the context of HFD-induced endothelial dysfunction. Because these phosphorylation measurements reflect whole-aorta signaling, endothelial-specific responses cannot be distinguished.

Considering that GRK2 is closely associated with clonidine-induced relaxation response (Taguchi et al., 2011; Taguchi et al., 2012b), we nest assessed GRK2 expression and activity in the aorta. GRK2 expression was elevated in HFD mice compared with SD mice, and FF administration had no effect (Figures 4A,E). GRK2 activity was also higher in HFD and HFD-FF mice compared with SD mice, although the differences were not statistically significant (Figure 4F). These results suggest that FF dose not influence GRK2 expression or activity in the aorta.

### 3.4 Fenofibrate inactivates hepatic GRK2

**FIGURE 5:**
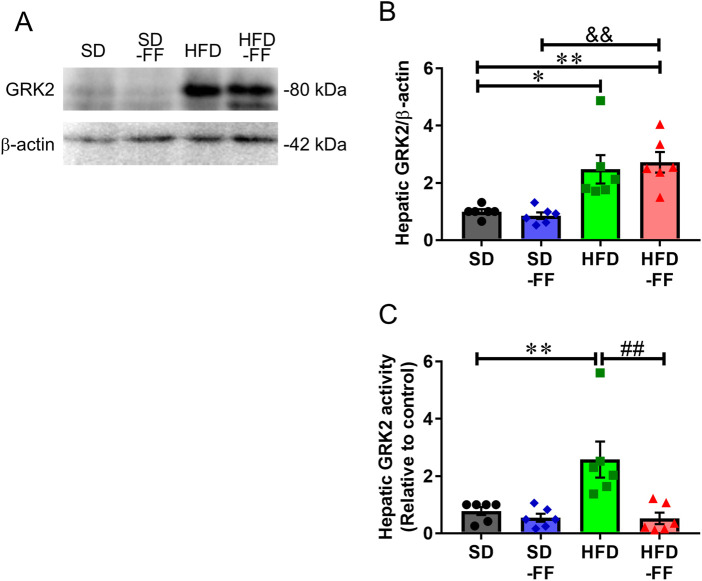
Effect of fenofibrate on the expression and activity of GRK2 in the liver. Protein levels were measured by Western blot assay in the livers of SD, SD-FF, HFD, and HFD-FF mice. **(A)** Representative Western blots, quantification of **(B)** GRK2 proteins, and **(C)** GRK2 activity (n = 6, mean ± SE). *p < 0.05, **p < 0.01 vs. SD. ^##^p < 0.01 vs. HFD. ^&&^p < 0.01 vs. SD-FF.

Because we previously reported that suppression of hepatic GRK2 expression and activity improves vascular endothelial reactivity (Taguchi et al., 2017), we next examined the effects of FF on hepatic GRK2. Hepatic GRK2 expression was increased in HFD and HFD-FF mice compared with SD mice, indicating that FF did not affect GRK2 expression (Figures 5A,B). However, hepatic GRK2 activity was markedly elevated in HFD mice compared with SD mice and markedly reduced in HFD-FF mice compared with HFD mice (Figure 5C).

## 4 Discussion

This study was designed to evaluate the effects of FF on endothelial function in dyslipidemia and to elucidate the potential signaling mechanisms involved. We employed a mouse model of HFD-induced dyslipidemia to investigate the effects of a 2-week treatment with FF (25 mg/kg/day) on endothelial dysfunction. Our results demonstrated that FF treatment reduced plasma and hepatic TG levels, whereas no significant differences were observed in plasma glucose or body weight between HFD mice and FF-treated HFD mice. Moreover, a 2-week FF treatment improved endothelial-dependent relaxation in the aorta and increased basal NO levels. Western blot analysis revealed that FF treatment enhanced LKB1 phosphorylation, leading to the activation of AMPKα and subsequent activation of Akt. These findings suggest that the vasorelaxation effects of FF are most likely mediated by modulation of the LKB1/AMPK/Akt pathway, resulting in increased NO production. We found that this beneficial effect was accompanied by suppression of GRK2 overactivation in the liver, but not in the aorta. Taken together, these findings indicate that FF improves lipid metabolism and HFD-induced endothelial dysfunction by enhancing the LKB1/AMPK/Akt signaling pathway and increasing NO production, while simultaneously suppressing hepatic GRK2 overactivation. These results provide new insights into the vasculoprotective mechanisms of FF in obesity-associated metabolic disorders.

Previous studies have shown that FF confers vasculometabolic benefits beyond lipid lowering, including anti-inflammatory effects relevant to endothelial protection (Staels et al., 1998; Lefebvre et al., 2006). However, the exact mechanisms remain unclear. Endothelial dysfunction is a primary driver of vascular complications in dyslipidemia, largely due to reduced NO production (Stehouwer, 2004). We found that FF treatment enhanced NO production in aortas from HFD mice. Endothelial-dependent relaxation and NO production induced by ACh and clonidine were impaired in aortas from HFD mice but were restored by FF. eNOS can be phosphorylated by AMPK (Chen et al., 1999; Morrow et al., 2003; Rodríguez et al., 2021; Hauger and Hordijk, 2024), and we observed increased AMPKα phosphorylation in aortas from FF-treated HFD mice. These findings align with earlier reports linking FF to AMPK activation (Jin et al., 2023; Kim et al., 2007; Tomizawa et al., 2011). Restoration of AMPK phosphorylation in HFD mice further supports the idea that AMPK is central to endothelial homeostasis under hemodynamic and metabolic stress conditions. Although the precise mechanism by which FF activates AMPK is not fully understood, prior studies have shown that LKB1 directly phosphorylates and activates AMPK as its principal upstream kinase (Shackelford and Shaw, 2009). Notably, our findings demonstrated that FF treatment enhanced the basal phosphorylation levels of LKB1, AMPK, and Akt in HFD mice, ultimately increasing eNOS phosphorylation and NO production. Although these changes are consistent with coordinated activation of the LKB1/AMPK/Akt axis, our data do not establish a direct causal hierarchy among these kinases. Thus, we interpret these signaling alterations as evidence of pathway involvement rather than a defined linear cascade. We attempted to assess eNOS phosphorylation at Ser1177 in aortic tissues; however, the basal (unstimulated) phosphorylation signal was extremely weak and technically inconsistent. Because all assays were conducted under similar basal conditions, we concluded that the incomplete eNOS phosphorylation data would not permit meaningful group comparisons and therefore excluded them from the analysis. Additional mechanistic studies will be required to directly evaluate eNOS activation and oxidative stress downstream of the LKB1/AMPK/Akt pathway. Consistent with this mechanism, we observed that FF enhanced LKB1, AMPK, and Akt phosphorylation in the aorta of HFD mice, accompanied by improved endothelial-dependent relaxation. Because LKB1 directly phosphorylates AMPK at Thr172 (Woods et al., 2003), the observed upregulation of LKB1/AMPK/Akt phosphorylation strongly suggests that FF restores endothelial NO production through reactivation of this canonical signaling axis. Functional vascular experiments further supported this interpretation. Preincubation of aortas from FF-treated HFD mice with an Akt inhibitor or Compound C (an AMPK inhibitor) attenuated the improvement in ACh- and clonidine-induced endothelial-dependent relaxation. This indicates that FF modulates vascular tone through AMPKα-related mechanisms, although additional studies are needed to clarify the specific steps involved. Together, these findings imply that FF stimulates the LKB1/AMPK/Akt pathway to enhance NO production, thereby improving endothelial-dependent relaxation under dyslipidemic conditions. The LKB1/AMPK/Akt axis serves as a crucial link between metabolic cues and eNOS activation, and our demonstration that FF restored this pathway fits within this established framework. FF did not substantially alter endothelial function or Akt-related signaling in SD mice, indicating that its beneficial actions are largely revealed under conditions of metabolic stress. Accordingly, the enhancement of clonidine-induced relaxation and Akt-dependent NO signaling appears to be HFD-dependent rather than a general effect in healthy vessels.The more pronounced inhibitory effect of the Akt blockade in SD aortas likely reflects the lower basal activation of the AMPK/Akt/eNOS pathway under physiological conditions. In contrast, because endothelial signaling is already attenuated in HFD vessels, the relative magnitude of inhibition produced by Akt inhibitor is smaller. Thus, the apparent difference in the inhibitor sensitivity between SD and HFD groups is consistent with differential basal pathway activity.

GRK2 is a serine/threonine kinase that regulates intracellular signaling of multiple G-protein coupled receptors (GPCRs) by phosphorylating activated receptors and promoting β-arrestin recruitment. Beyond receptor desensitization, GRK2 also modulates intracellular signaling networks. Interaction between GRK2 and Akt has been shown to suppress Akt kinase activity, contributing to endothelial dysfunction in diabetes and metabolic disorders (Taguchi et al., 2011; Taguchi et al., 2012a; Taguchi et al., 2012b; Taguchi et al., 2015; Taguchi et al., 2017; Gurevich et al., 2016; Bencivenga et al., 2021). Elevated GRK2 levels have been reported in vascular tissues under insulin resistance and hyperglycemia, where they impair eNOS activation and vascular relaxation (Taguchi et al., 2011; Taguchi et al., 2012a; Taguchi et al., 2012b; Taguchi et al., 2017). Excessive GRK2 activity is, therefore, considered a key contributor to endothelial dysfunction and insulin resistance (Ciccarelli et al., 2011), positioning GRK2 as a molecular bridge between metabolic dysregulation and vascular pathology. Consistent with previous findings, we observed increased GRK2 expression and activation in the vasculature of HFD mice. However, FF did not alter GRK2 expression or activity in the vasculature, suggesting that its vascular benefits occur independently of GRK2 modulation in blood vessels. In contrast, FF markedly reduced hepatic GRK2 activity, indicating tissue-specific regulation of GRK2 between liver and vascular tissues. This observation aligns with evidence that hepatic GRK2 plays a pivotal role in systemic metabolic regulation. Notably, our previous work showed that selective inhibition of hepatic GRK2 using liver-targeted siRNA in diabetic mice was sufficient to restore endothelial-dependent relaxation and Akt/eNOS signaling without altering vascular GRK2 expression (Taguchi et al., 2017), establishing a causal liver–vascular axis mediated by hepatic GRK2. Although liver-specific GRK2 modulation was not performed in the present HFD model, the observed pattern of hepatic GRK2 overactivation and its suppression by FF suggest that a similar mechanism may contribute to the vascular improvements reported here. Future studies employing liver-specific GRK2 modulation in HFD mice will be necessary to directly test this hypothesis. For example, inducible deletion of GRK2 in mice reverses diet-induced obesity and insulin resistance, mitigates hepatic steatosis, and improves insulin sensitivity (Vila-Bedmar et al., 2015). Similarly, GRK2 downregulation enhances insulin signaling and lipid metabolism in liver and adipose tissue, underscoring its function as a key regulator of metabolic homeostasis (Sorriento et al., 2021). Our currents demonstrate that FF suppressed hepatic GRK2 activity but not alter vascular GRK2, suggesting that hepatic GRK2 plays a more dominant role in systemic lipid metabolism and its downstream vascular effects. This interpretation is supported by prior reports showing that hepatic GRK2 suppression improves glucose metabolism and endothelial function (Taguchi et al., 2017). Improvements in lipid metabolism have also been shown to ameliorate vascular endothelial dysfunction (Miller et al., 2013; Xu et al., 2019; Wang et al., 2025). Thus, hepatic GRK2 modulation may contribute indirectly to vascular protection by restoring systemic metabolic balance. Although our findings support a model in which FF improves endothelial function primarily via metabolic improvement driven by the suppression of hepatic GRK2 activity, we cannot entirely exclude the possibility of direct effects on vascular endothelial cells through PPARα/AMPK signaling. FF activates AMPK in vascular tissues and enhances endothelial responses independently of systemic lipid regulation (Kim et al., 2007; Tomizawa et al., 2011). In the present study, vascular PPARα expression was detectable but did not differ among the four groups (data provided in the response document), suggesting that FF-induced restoration of endothelial function in HFD mice is unlikely to result from enhanced vascular PPARα signaling. Nevertheless, *ex vivo* experiments or cell-type–specific PPARα activation studies will be required to definitively distinguish direct vascular action from liver-derived systemic contributions. Moreover, although GRK2 expression was elevated in the aorta of HFD mice, FF did not alter vascular GRK2 expression or activity, indicating that vascular GRK2 is unlikely to mediate the endothelial improvements. Instead, FF markedly suppressed hepatic GRK2 activation, concomitant with improved systemic lipid metabolism. These findings suggest that the vascular benefits of FF are more plausibly attributed to metabolic normalization via reduced hepatic GRK2 activity rather than direct GRK2 modulation with the vasculature.

From a translational standpoint, our findings highlight hepatic GRK2 as a potential therapeutic target linking metabolic regulation to vascular homeostasis. Strategies aimed at modulating hepatic GRK2 activity and restoring the LKB1/AMPK/Akt/eNOS pathway may provide novel opportunities to ameliorate endothelial dysfunction in obesity-related cardiometabolic diseases. However, several limitations should be acknowledged. First, this study was conducted exclusively in male ICR mice, and potential gender-based differences in endothelial dysfunction or vascular responses to FF were not assessed. Future studies including both sexes will be important, given known sex-related variation in endothelial NO signaling. Second, although HFD is often associated with insulin resistance, we did not directly measure fasting glucose, insulin, or insulin sensitivity; therefore, the metabolic phenotype of this model is interpreted primarily as obesity/dyslipidemia rather than insulin resistance. Third, FF was administered via short-term intraperitoneal injections, which differs from the chronic oral administration used clinically. Future studies evaluating longer-term dosing regimens will be necessary to evaluate translational relevance. In addition, resistance arteries, where endothelial dysfunction is typically more pronounced in obesity, were not assessed. The thoracic aorta was selected because NO-dependent vasorelaxation a key mechanism underlying FF’s effects can be reliably measured in this vessel. Future studies using small arteries will help determine whether the hepatic GRK2–vascular axis extends to resistance vascular beds. Finally, phosphorylation levels of AMPK and Akt were measured using whole aortic homogenates, representing mixed endothelial and smooth muscle signaling. Although the functional improvements such as ACh- and clonidine-induced relaxation (with preserved SNP responses) strongly suggest endothelial enhancement, additional studies using purified endothelial cell or endothelial-specific approaches will be required to delineate cell-type–specific mechanisms.

In summary, this study demonstrates that FF ameliorates vascular endothelial dysfunction in HFD-mice primarily through the restoration of systemic lipid metabolism. Importantly, FF reduced hepatic GRK2 activity, which contributes to the normalization of metabolic homeostasis. This metabolic improvement was accompanied by enhanced endothelial NO production through activation of the LKB1/AMPK/Akt/eNOS pathway. Thus, although FF did not directly alter vascular GRK2 expression or activity, its suppression of hepatic GRK2 may represent an important mechanism by which FF improves lipid metabolism and secondarily preserves vascular endothelial function in obesity. Taken together, these findings highlight the therapeutic potential of targeting hepatic GRK2 as a means to correct metabolic abnormalities and protect vascular health in obesity-related CVDs.

## Data Availability

The original contributions presented in the study are included in the article, further inquiries can be directed to the corresponding author.
